# Peripheral Arterial Disease and the Diabetic Foot Syndrome: Neuropathy Makes the Difference! A Narrative Review

**DOI:** 10.3390/jcm13072141

**Published:** 2024-04-08

**Authors:** Gerhard Rümenapf, Nour Abilmona, Stephan Morbach, Martin Sigl

**Affiliations:** 1 Department of Vascular Surgery, Deaconess Foundation Hospital, 67346 Speyer, Germany; nour.abilmona@diakonissen.de; 2 Department of Diabetology and Angiology, Marien-Krankenhaus, 59494 Soest, Germany; s.morbach@hospitalverbund.de; 3 Division of Angiology, First Department of Medicine, Faculty of Medicine of the University of Heidelberg, University Medical Center Mannheim UMM, 68167 Mannheim, Germany; martin.sigl@umm.de

**Keywords:** critical limb-threatening ischemia, CLTI, diabetic foot syndrome, DFS, neuropathy, nociception, peripheral arterial disease, PAD, revascularization

## Abstract

**Background**: In vascular medicine, peripheral arterial disease (PAD) and diabetic foot syndrome (DFS) are often considered synonymous with respect to the need for revascularization. In PAD patients, clinical symptoms reflect the degree of atherosclerotic disease, since peripheral innervation, including pain sensation, is not usually compromised. In DFS patients, however, symptoms of relevant foot ischemia are often absent and progression of ischemia goes unnoticed owing to diabetic polyneuropathy, the loss of nociception being the main trigger for foot ulcers. This review analyzes the fundamental differences between PAD and DFS against the background of polyneuropathy. **Methods**: The literature research for the 2014 revision of the German evidence-based S3-PAD-guidelines was extended to 2023. **Results**: Vascular examination is imperative for both, PAD and DFS. Stage-dependent revascularization is of utmost importance in PAD patients, especially those suffering from critical limb-threatening ischemia (CLTI). Successful therapy of DFS goes further, including infection and metabolic control, wound management, offloading the foot and lifelong prophylaxis in the course of a multidisciplinary treatment concept. Revascularization is not needed in all cases of DFS. **Conclusions**: There are fundamental differences between PAD and DFS with respect to pathophysiology, the anatomical distribution of arterial occlusive processes, the clinical symptoms, the value of diagnostic tools such as the ankle-brachial index, and classification. Also, therapeutic concepts differ substantially between the two patient populations.

## 1. Introduction

Peripheral arterial disease (PAD) and diabetes mellitus (DM) are increasingly common worldwide [1,2,3]. PAD leads to atherosclerotic occlusions of leg arteries, with symptoms ranging from walking pain to painful tissue loss, occasionally ending in major amputation.

Patients with DM are prone to developing painless foot lesions (diabetes-related foot syndrome, DFS) owing to diabetic polyneuropathy (PNP). Similar foot lesions have long been known in patients with syphilis, leprosy, or alcoholism, the common denominator being the loss of protective sensation nociception, [4] in combination with mechanical triggers [5,6,7]. In addition, DFS patients are at high risk for PAD [6], and, in turn, DFS patients with PAD are at a much higher risk for non-healing foot wounds and amputation than PAD patients without neuropathy [8,9].

In vascular medicine, PAD and DFS are often considered equivalent with respect to the need for revascularization, although PAD is not the dominant cause of DFS foot lesions [5,7,8,9]. Stage-appropriate revascularization has absolute priority in PAD [10,11] but in the complex, interdisciplinary treatment of DFS, it is not required in all cases.

In this review, we analyze the similarities and differences between “PAD without neuropathy” and “PAD with neuropathy” (e.g., DFS) with regard to pathophysiology, diagnostics, therapy, and aftercare.

## 2. Methods

The German Society for Angiology (DGA) performed an extensive literature search for the 2014 edition of its evidence-based PAD guidelines [11]. For the present article, that particular literature search was extended to 2023 using MEDLINE, PubMed, and the Cochrane Database of Systemic Reviews. Search items comprised CLTI, chronic limb-threatening ischemia, diabetic foot syndrome, DFS, peripheral arterial disease, PAD, diabetic polyneuropathy, revascularization. Individual publications were checked for previously unidentified studies. Particular importance was assigned to randomized controlled trials (RCTs) and their meta-analyses.

## 3. Epidemiology

PAD affects more than 250 million people worldwide. It is caused by atherosclerotic occlusion of the pelvic and leg arteries [1,2]. More than 20% of people over the age of 65 suffer from PAD [12]. PAD is globally underrated, underdiagnosed, and undertreated [13]. The presence of PAD indicates concomitant cardiac and cerebrovascular arterial disease and predicts a reduced life expectancy [14]. One-third of PAD patients have clinical symptoms, mostly pain during walking (intermittent claudication, CI). A total of one in 100 patients per year [10,11] develops critical limb-threatening ischemia (CLTI). The risk of amputation increases dramatically in the case of concomitant DM.

The global number of people with DM currently exceeds 500 million [3]. In Germany, DM affects more than 10% of the population [15], while the overall incidence of diabetes mellitus type 2 is decreasing in high-income countries [16,17]. DFS is a lifelong complication of DM, consisting of active phases and remissions (inactive phases) and having a high recurrence rate [7,18,19]. 

In a recent 5-year follow-up study on patients with diabetes, the initial prevalence of diabetic neuropathy was up to 36%, especially in patients with severe insulin-deficient diabetes [20]. Diabetic polyneuropathy affects approximately 50% of adults with diabetes during their lifetime [21]. Approximately 10–30% of them have symptomatic neuropathy with pain or other discomfort. However, the loss of nociception (due to degeneration of non-myelinated afferent A-delta and C-fibers) mostly goes unnoticed for the patient and the investigator. It is present in nearly all patients with diabetic foot lesions [4], or in other words, a painless foot ulcer in a diabetic patient is proof of the neuropathic loss of nociception. 

In addition to PNP, micro- and macrovascular disorders, tissue edema, and septic thrombosis following local infections in the presence of an impaired immune defense [22] all contribute to DFS. The prevalence of foot lesions is about 3% in diabetic patients [23], and 25% of these patients will suffer from DFS in their lifetime, the incidence being more than 2% [7]. DFS is the most common cause (about 70%) of major limb amputations, of which about 10,000 are performed annually in patients with DFS in Germany [24]. The relative risk of a major amputation in people with DM remains to be more than five times that of non-diabetics [25,26].

## 4. Pathophysiology and Clinic

In sole PAD, the iliac and femoral arteries are predominantly affected. Symptoms (Table 1) correlate with the severity of atherosclerotic occlusive disease and the ankle–brachial index (ABI; see below). Pain caused by ischemia, injury, or abnormal pressure gives an early warning. Thus, PAD is usually identified and treated before tissue loss occurs. Painful ischemic neuropathy may accompany CLTI. The risk of amputation in PAD without PNP is lower than that in DFS [10,11,27].

DFS consists of “foot ulceration, infection, or tissue destruction associated with pre-existing neurological disorders and/or PAD” in the presence of diminished immune response [28]. In Germany, callus and rhagades are included in the definition of DFS [29].

**Figure 1:**
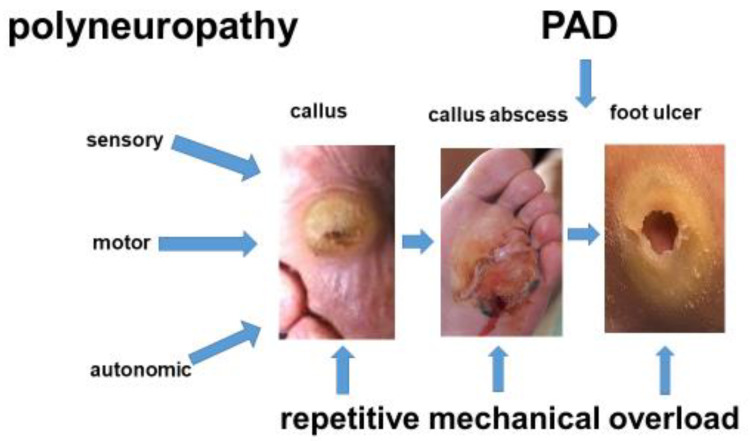
Diabetic foot ulcers usually result from repetitive pressure overload in the presence of polyneuropathy (PNP). First, a painless, abnormally thick callus develops, followed by an underlying hematoma. This may lead to a callus abscess, which breaks open (“mal perforant”). In the case of relevant peripheral arterial disease (PAD), wound healing stagnates despite pressure relief and stage-appropriate wound treatment. The patient then requires additional treatment for PAD, despite PNP being the primary cause of the foot lesions.

The pathophysiology of DFS is more complex than that of PAD. Most patients with DFS have a loss of protective sensation (LOPS). Usually, 50% of these patients have relevant PAD [6], whereas CLTI goes unnoticed until foot lesions with inadequately low pain occur. These foot lesions do not heal despite pressure relief and systematic wound treatment (Figure 1). In 70% of cases, the lower leg arteries are primarily affected, while the arteries of the foot are often spared [30]. While the deep femoral artery is often affected by occlusive processes in DFS patients, the superficial femoral and popliteal artery are less commonly affected than in PAD patients without neuropathy [31].

Ischemic foot lesions in DM without PNP are rare and not defined as DFS because the trigger of foot lesions, neuropathy, is absent.

Foot lesions similar to those seen in DFS patients today have long been observed in patients with syphilis, leprosy, or alcoholism. The common denominator in these diseases is polyneuropathy (Table 2). In DFS, neuropathy is associated with diabetic microangiopathy [32], see below, which presumably starts long before disturbed glucose metabolism is revealed [33]. Other factors, such as the duration of diabetes, the quality of glycemic control, deficient insulin function, etc., have all been identified to contribute to diabetic PNP [34]. Sensory PNP leads to deficits in pressure, pain, and temperature perception. DFS patients walking with painless foot ulcers [4,5] prove to have a loss of non-myelinated nociceptive C-fibres, while other sensory qualities may still be present [4].

Motor neuropathy leads to crural–pedal muscle atrophy. The preponderance of the calf muscles over the foot dorsiflexors causes bunions and equinus [5,7]. Atrophy of the plantar fat increases the pressure load on the metatarsal heads. Hammer and claw toes, predilection sites for pressure sores, develop from drop foot compensation by the toe lifter muscles.

Autonomous neuropathy leads to precapillary AV shunting “auto-sympathectomy” [35]. Thereafter, chronic capillary ischemia of deep foot tissues ensues, independently from PAD. The skin is warm and rosy, cracked, and scaly, as sweating is reduced.

**Figure 2:**
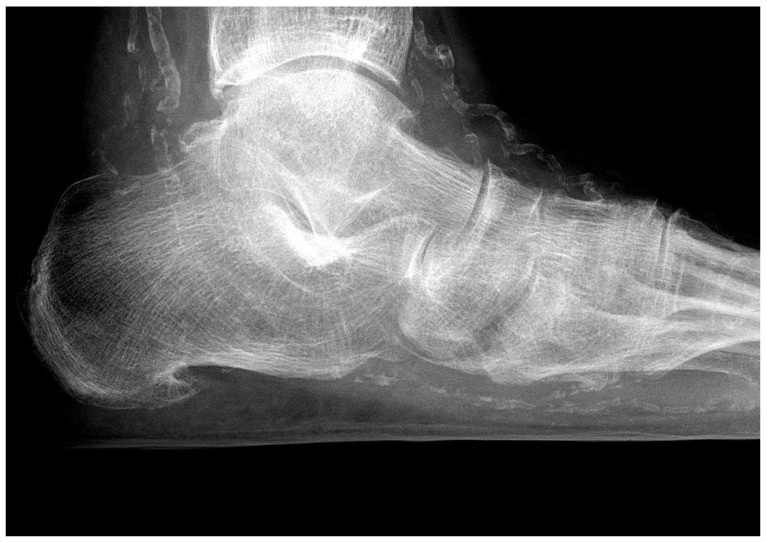
Media sclerosis of the pedal arteries in a patient with extensive polyneuropathic foot ulcers. HbA1C is 4.8%, and the origin of PNP is chronic alcohol abuse.

Additional factors compromise blood flow to foot tissues in patients with DFS, impair wound healing, and make PAD more threatening than in people without diabetes (Figure 2). Diabetic microangiopathy impairs oxygen diffusion to the surrounding tissue due to the thickening of the capillary basement membrane. Additionally, PNP is responsible for mediasclerosis of the infrapopliteal arteries. Consequently, vessel wall elasticity is reduced, oxygen supply to the tissue is impeded, and the development of distal PAD is accelerated [36]. The incompressibility of the vessel wall is responsible for an incorrectly high ankle–brachial index (ABI) in many DFS patients, making its use as a diagnostic tool for the estimation of arterial perfusion questionable in these patients.

Interestingly, non-diabetic patients with a polyneuropathic (Figure 2) loss of nociception and foot ulcers demonstrate similar clinical and radiological features as neuropathic diabetic patients. This underlines the key role of peripheral polyneuropathy in the development of foot lesions.

### 4.1. Skeletal Problems

**Figure 3:**
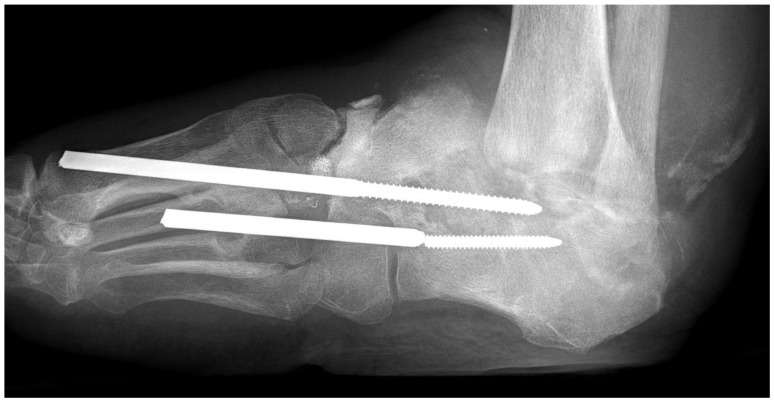
Charcot foot with destruction of the hindfoot and ankle joints in a diabetic patient without PAD but with maximal polyneuropathy. Because of ignorance of the pathophysiology, osteosynthetic experimental surgery aimed at stabilizing the longitudinal arch of the foot has been performed.

In PAD without neuropathy, pathological fractures of the foot skeleton are rare. On the other hand, patients with DFS may develop neuropathic osteo-arthropathy (DNOAP, Charcot foot), a dystrophic degeneration of the foot skeleton with deformities. Most evidently, the combination of mechanical overload and the loss of trophic bone innervation, pain sensation, and nociceptor function lead to bone loss and painless pathological fractures (Figure 3) in cases of gross deformity often combined with plantar ulcers.

### 4.2. Neuropsychiatric Disturbances

PAD patients with CLTI usually present with pain and unhealthy dependencies, such as excessive cigarette smoking or alcohol abuse, reflecting a low socio-economic status. DFS patients are inclined to develop endogenous depression and ignore metabolic control of diabetes. Due to PNP-related loss of afferences, they cease to regard their feet as part of their body (asomatognosia), and, therefore, do not adequately care for them (neglect).

### 4.3. Immunodeficiency

Hyperglycemia in diabetes leads to a dysfunction of the immune response. This facilitates the propagation and spreading of invading pathogens in diabetic patients, making them more prone to infections, such as phlegmons of the foot, as seen in DFS [22].

### 4.4. Diagnosis of PAD

Regardless of the coexistence of diabetes, PAD is diagnosed in the following order [10,11]:

- Inspection of the legs and feet;
- Bilateral palpation of the femoral, popliteal, ankle, and foot pulses;
- Doppler sonographic measurement of the ankle–brachial index (ABI) or toe–brachial index (TBI);
- Duplex sonography of the pelvic and leg arteries;
- Digital diagnostic/therapeutic subtraction angiography (DSA);
- MRA and CTA.

The measurement of the foot skin temperature using infrared thermography has become a standard in the clinical investigation of a DFS patient with foot lesions. Increased temperature is a strong indicator of imminent tissue damage, so this tool can reduce the risk of diabetic foot ulceration [37,38]. However, owing to autonomous neuropathy, skin temperature may be falsely high despite CLTI, while in PAD patients, the skin temperature is usually low (Table 3).

DFS patients often have PNP-related foot and toe deformities (Table 3), which facilitate the development of foot ulcers due to pathologically high-pressure loads [5]. This is rare with PAD alone. DFS patients may have severe foot lesions in the absence of macroangiopathy, while ulcers in PAD patients directly reflect the degree of foot ischemia.

In PAD patients, the iliac and femoral arteries are predominantly affected, reflected by the absence of femoral or popliteal pulses (Table 4). In patients with DFS, the popliteal pulse is often palpable, with the crural arteries being affected in about 70% of cases [30]. Therefore, in these DFS patients, a DSA of the crural arteries can be performed in readiness for intervention.

While the ankle–brachial index (ABI) (Table 4) reliably reflects the degree of ischemia in PAD, it falls short in most DFS patients. Only an ABI below 0.6 may indicate CLTI [39]. Higher ABI values may be unreliable because media sclerosis leads to falsely high ABI values, overestimating the true arterial blood pressure. Therefore, CLTI is often overlooked in DFS patients. Occlusion pressures of the toe arteries (often spared by mediasclerosis) may be more reliable (toe–brachial index, TBI [40]).

## 5. Classifications

Staging of PAD (Table 1) is based on pain and tissue loss, reflecting the extent of PAD and the urgency of revascularization.

With DFS, CLTI often goes unnoticed due to concomitant PNP, making its staging along PAD criteria questionable, as long as the extent of PAD is suspected only by the extent of tissue damage (Table 4). Serious damage to the foot of diabetic patients may erroneously be taken as the expression of CLTI and can lead to hasty decisions for major amputation. CLTI with massive tissue loss in DFS may have the same bad prognosis as the Rutherford stages V and IV, but the pathophysiology is fundamentally different, and, therefore, in our opinion, the PAD classification is not adequate for DFS. In other words, the Rutherford classification can serve only when the child has already fallen into the well. In only half of the DFS patients presenting with foot lesions, PAD is not the most important factor.

In Germany [29], the Wagner classification is used as the standard for grading diabetic foot lesions with respect to their depth and extent. It is combined with the University of Texas classification, which includes the two criteria: infection and ischemia [29]. However, its information on pathophysiology is poor since it does not provide information on whether diabetic PNP is present or not. More appropriate seems the SINBAD classification [41,42], which includes localization (S, site), ischemia (I), neuropathy (N), bacterial infection (B), extent (A, area), and depth (D, depth) of the ulcer.

The WIfI System [36] assesses the risk of amputation and the prognosis of CLTI in both PAD patients and DFS patients with concomitant PAD (Table 4). It includes the three criteria: wound, ischemia, and foot infection (WIfI). Healing times and amputation rates correlate with WIfI severity levels. The criterion “neuropathy”, however, is missing from this classification system. WIfI is recommended for assessing the benefit of revascularization [43] and is primarily used scientifically.

## 6. Therapy

All therapeutic measures for PAD and DFS (Table 5) are based on lifestyle changes, smoking cessation, and treating risk factors [10,11]. Both, timely vascular diagnostics and revascularization have physical and psychological advantages for the affected patients and are cost-effective for the healthcare system.

PAD: The aim of treatment is to improve arterial blood flow by walking exercise (SET, supervised exercise training), endovascular interventions, or open surgical procedures. The focus of treatment is the improvement of the quality of life and, more importantly, the preservation of the lower limbs. The chances of success are high, especially if the patients are willing to undergo lifestyle modifications [44].

With intermittent claudication, the pain-free walking distance can be doubled within a year with SET [44,45]. Both, less supervised or non-supervised walking exercise, on the other hand, have no significant effect on the pain-free walking distance in these patients [45]. Revascularization should be considered if conservative treatment was not successful, in case of severe occupational limitations, or restrictions in daily life [10,11].

DFS: The complex pathophysiology of PAD with PNP (mostly DFS) requires a multidisciplinary treatment strategy [46]. This can reduce the amputation rate by approximately 80%. Pressure relief (offloading), stage-appropriate wound treatment with regular wound debridement, and antibiotic therapy in case of infection are the basic measures. In case of concomitant PAD, endovascular or vascular surgical options for revascularization should be considered early. Physical training has a positive influence on blood sugar control and cardiac comorbidities, as well as microangiopathic complications and neuro-musculo-skeletal deficits [45,47]. However, if physical activity leads to an overload of the feet (plantar tissue stress) in the presence of PNP, the risk of foot ulcers increases despite adequate shoe care.

There is no evidence for a benefit of SET (recorded with a treadmill) in DFS patients [48,49] since ischemia-related pain (intermittent claudication) is absent due to PNP. “Non-weight-bearing” training has been recommended for these patients, but there is no evidence for this recommendation either [48]. In addition, in DFS, a crural–pedal arterial occlusion pattern prevails, where diabetic PNP is most pronounced. The International Working Group on the Diabetic Foot (IWGDF) recommends that DFS patients with a low risk of developing a foot ulcer walk up to 1000 more steps per day with optimal footwear [50], which, unfortunately, does not correspond to the reality of care for all those affected.

## 7. Revascularization

Symptomatic (pain, gangrene) PAD patients have the chance for revascularization much earlier than people with diabetic polyneuropathy who do not usually present until tissue loss has already occurred.

The indications for vascular surgery and endovascular procedures are identical for both groups, patients with PAD and patients with DFS. Endovascular treatment should be preferred for all vascular levels and vascular stages [10,11] due to having lower rates of trauma, periprocedural morbidity and mortality, infections, and wound healing disorders, in comparison to open vascular surgery. In CLTI, the results of bypass surgery using an autologous vein are significantly better than those of endovascular therapy. The difference disappears when synthetic prostheses are used as bypass material [51,52], or may even favour endovascular therapy in patients who no longer have a vein as a bypass vessel [53]. Prosthetic bypasses should be regarded as the last resort before major amputation, owing to the risk of infection considering the diabetic patients’ immunodeficiency.

Vascular medical care in DFS requires in particular infrapopliteal measures. Despite better long-term patency rates for infrapopliteal bypass grafts compared to endovascular procedures [54] in CLTI, ulcer healing rates, amputation rates, and foot-sparing rates are the same for both procedures [40,55]. Neither method is superior to the other. The method of revascularization should depend on the length of the occlusion, especially of the crural arteries, the presence of a suitable bypass vein, the patient’s prognosis quoad vitam, and the equipment available, as well as the expertise of the surgeon/interventionalist [40]. The results of bypass surgery are equally good in patients with and without DM, but mortality and the risk of amputation are higher in DM [55], as is the risk of myocardial infarctions, cardiac arrhythmias, heart failure, wound infections, and renal failure [56].

## 8. Recurrence and Follow-Up

PAD: Following successful revascularization, a decrease of the pain-free walking distance or the recurrence of painful foot ulcers or gangrene usually indicates a failure of the arterial reconstruction. This occurs after femoral patchplasty in about 20%, after femoro-popliteal vein bypass in 30% of cases after 5 years. After endovascular revascularization, restenosis rates are significantly higher [10,11]. In the long term, new atherogenic occlusion processes in upstream or downstream vascular regions can also explain the failure of the arterial reconstruction. For PAD patients with bypasses, regular duplex sonographic checks of the flow rate may help prevent imminent bypass occlusion. Following endovascular revascularization, sonographic follow-up is not useful until reoccurence of symptoms.

DFS: Diabetic foot lesions recur in more than 30% of the patients one year after a previous lesion and in over 70% of patients after 5 years, which is a much higher recurrence rate than that in patients with PAD without PNP [48]. Owing to diabetic PNP, lesions will recur despite optimal arterial reconstruction due to pathological pressure loads in unsuitable footwear in combination with foot and toe deformities. The primary task of recurrence prevention is, therefore, regular medical examination of feet and shoes at 3- to 6-month intervals, as well as continuous reminders for patients to regularly self-check their feet and reduce the walking distance [28,29]. In this context, surgical correction of toe deformities (e.g., by minimally invasive tenotomy) as well as of ball and equinus deformities of the foot (e.g., by gastrocnemius release [5,7]) helps prevent recurrent lesions on the feet.

Regular ultrasound monitoring is mandatory in DFS patients following surgical or endovascular revascularization since failure of arterial reconstructions, such as bypass or stent occlusions, may go unnoticed, owing to PNP, and wounds may deteriorate without any symptoms.

## 9. Conclusions for Practice

1. Mechanical overload in PAD patients is painful. In DFS patients, it leads to painless foot ulcers;
2. In PAD, the degree of ischemia is reflected by the ankle–brachial index (ABI). In DFS, the ABI is misleading, owing to PNP-driven mediasclerosis of the infrapopliteal arteries;
3. Tissue loss in PAD reflects CLTI, while foot lesions in DFS primarily indicate PNP. Since PNP may mask CLTI, assessment of the arterial perfusion of the legs is mandatory for both patient groups;
4. In PAD, occlusive processes are localized more proximally (pelvic, femoral arteries) than in neuroischemic DFS (crural–pedal arteries). PNP-related vascular pathologies (media sclerosis, chronic capillary ischemia) additionally reduce the blood flow to the feet in DFS patients;
5. PAD and DFS represent fundamentally different pathophysiological entities. Hence, therapeutic concepts differ substantially between the two patient populations;
6. Revascularization is crucial in PAD patients. In DFS patients, offloading and stage-appropriate wound care predominate the interdisciplinary treatment concept. Revascularization is mandatory if coexisting PAD prevents the healing of foot lesions;
7. Classifying DFS patients according to the degree of ischemia (Fontaine, Rutherford) may be misleading since ABI measurement is not reliable, and the criterion “pain” is missing.

## Figures and Tables

**Table 1:** Classification of peripheral arterial disease (PAD) according to Fontaine and Rutherford.

Fontaine		Rutherford		
stage	symptoms	grade	category	symptoms
I	asymptomatic	0	0	asymptomatic
II a	walking distance > 200 m	I	1	marginal
II b	walking distance < 200 m	I	2	significant
		I	3	serious claudication
III	ischemic rest pain	II	4	ischemic rest pain
IV	tissue loss	III III	5 6	small area necrosis extensive necrosis

**Table 2:** Synopsis 1: Differences between peripheral arterial disease (PAD) without neuropathy and diabetic foot syndrome (DFS). Neurological, neuropsychiatric, and immunological aspects.

	PAD without Neuropathy	PAD with Neuropathy (Mostly DFS)
wound pain	high	low
CLTI-related pain level	high	low
loss of protective sensation (LOPS)	rare (alcohol abuse)	standard
activity level	reduced (pain)	inadequately high
motor neuropathy	none	frequent
ischemic neuropathy	rarely	none
autosympathectomy	no	frequent
clinical sing of overload	pain	foot ulcer
skeletal changes	rare	frequent (Charcot foot)
main cause of foot lesions	CLTI	neuropathy pressure overload foot/toe deformities
neuropsychiatric problems	dementia (age-dependent) alcohol abuse (frequently)	depression, neglect, loss of body perception (asomatognosia)
Immunodeficiency	rare	frequent

**Table 3:** Synopsis 2: Differences between PAD patients without neuropathy and DFS patients. Inspection and physical examination of the feet.

	PAD without Neuropathy	PAD with Neuropathy (Mostly DFS)
soft tissue edema	rare (heart failure)	common
toenails	-	frequently mycotic
skin of the foot	atrophic, thin, cold, paling when elevated	dry, warm, rosy, filling of veins even when elevated
skin of the foot sole	atrophy without hyperkeratosis	hyperkeratosis, calluses, fissures, pressure ulcers
pedal muscles	-	commonly atrophic
plantar fat pad	-	atrophic
foot position	normal	ball foot, pointed foot (shortening of calf muscles)
toes	no hair, livid acral lesions	claws/hammer toes, corns
localization of foot lesions	indicates areas without sufficient residual blood flow	reveals pathobiomechanics
neurological deficits	rare (ischemic neuropathy)	common (pain, temperature, vibration, painful painless foot etc.)
infrared thermography	low skin temperature	high skin temperature may favor overestimation of arterial perfusion

**Table 4:** Synopsis 3: Differences between PAD without neuropathy and DFS: vascular aspects and classification.

	PAD without Neuropathy	PAD with Neuropathy (Mostly DFS)
affected vessels	macroangiopathy	microangiopathy, macroangiopathy
microagiopathy-related impaired oxygen diffusion	no	common
chronic capillary ischemia	no	common
media sclerosis	no	yes
distribution of PAD	Iliaco-femoral	infrapopliteal or pedal
multilevel disease	common	common
popliteal pulse	frequently lost	often palpable
pain	strong	weak or missing
ankle–brachial index (ABI)	useful	useless (falsely high)
toe–brachial index (TBI)	unnecessary	useful
staging according to Fontaine or Rutherford	useful	questionable
classification (e.g., SINBAD)	useless	useful
WIfI classification	useful	useful

**Table 5:** Synopsis 4: Differences between PAD without neuropathy and DFS with respect to treatment and aftercare.

	PAD without Neuropathy	PAD with Neuropathy (Mostly DFS)
treatment concept	vascular	multidisciplinary
timely treatment of CLTI	common	usually too late
supervised walking exercise training (SET)	IC: useful CLTI: dangerous	dangerous
revascularization solves the problem	mostly	rarely alone
offloading performed by the patient	mostly (pain)	rarely (neuropathy)
recurrence rate of ulcers/necroses	low	high
ultrasound bypass control	yes	yes
ultrasound control of endovascular reconstructions	no	regularly
